# Connectome gradient dysfunction contributes to white matter hyperintensity‐related cognitive decline

**DOI:** 10.1111/cns.14843

**Published:** 2024-07-12

**Authors:** Dan Yang, Yi Tan, ZhiXin Zhou, Zhihong Ke, Lili Huang, Yuting Mo, Limoran Tang, ChengLu Mao, Zheqi Hu, Yue Cheng, Pengfei Shao, Bing Zhang, Xiaolei Zhu, Yun Xu

**Affiliations:** ^1^ Department of Neurology, Nanjing Drum Tower Hospital Clinical College of Nanjing Medical University Nanjing China; ^2^ Department of Neurology, Nanjing Drum Tower Hospital, Affiliated Hospital of Medical School Nanjing University Nanjing China; ^3^ Department of Radiology Affiliated Drum Tower Hospital of Nanjing University Medical School Nanjing China; ^4^ Department of Neurology, Nanjing Drum Tower Hospital, State Key Laboratory of Pharmaceutical Biotechnology and Institute of Translational Medicine for Brain Critical Diseases Nanjing University Nanjing China

**Keywords:** cognitive decline, executive function, functional connectome gradient, white matter hyperintensity

## Abstract

**Background:**

Although white matter hyperintensity (WMH) is closely associated with cognitive decline, the precise neurobiological mechanisms underlying this relationship are not fully elucidated. Connectome studies have identified a primary‐to‐transmodal gradient in functional brain networks that support the spectrum from sensation to cognition. However, whether connectome gradient structure is altered as WMH progresses and how this alteration is associated with WMH‐related cognitive decline remain unknown.

**Methods:**

A total of 758 WMH individuals completed cognitive assessment and resting‐state functional MRI (rs‐fMRI). The functional connectome gradient was reconstructed based on rs‐fMRI by using a gradient decomposition framework. Interrelations among the spatial distribution of WMH, functional gradient measures, and specific cognitive domains were explored.

**Results:**

As the WMH volume increased, the executive function (*r* = −0.135, *p* = 0.001) and information‐processing speed (*r* = −0.224, *p* = 0.001) became poorer, the gradient range (*r* = −0.099, *p* = 0.006), and variance (*r* = −0.121, *p* < 0.001) of the primary‐to‐transmodal gradient reduced. A narrower gradient range (*r* = 0.131, *p* = 0.001) and a smaller gradient variance (*r* = 0.136, *p* = 0.001) corresponded to a poorer executive function. In particular, the relationship between the frontal/occipital WMH and executive function was partly mediated by gradient range/variance of the primary‐to‐transmodal gradient.

**Conclusions:**

These findings indicated that WMH volume, the primary‐to‐transmodal gradient, and cognition were interrelated. The detrimental effect of the frontal/occipital WMH on executive function was partly mediated by the decreased differentiation of the connectivity pattern between the primary and transmodal areas.

## Graphical abstract

The WMH volume, primary‐to‐transmodal gradient, and cognition were interrelated. The detrimental effect of the frontal/occipital WMH on executive function was mediated by the decreased differentiation of the connectivity pattern between the primary and transmodal areas.

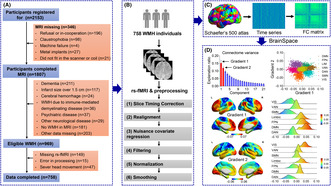

## 1 INTRODUCTION

White matter hyperintensity (WMH), which presents as hyperintense of subcortical white matter on T2‐weighted imaging (T2WI) or fluid‐attenuated inversion recovery (FLAIR) imaging, is a cardinal manifestation of cerebral small vessel disease (CSVD) frequently observed in elderly individuals.^1^ It has been reported that 72%–96% of people over 60 years old and nearly 100% of people over 90 years old have WMH^2^, ^3^ and that the prevalence and severity of WMH increase with age.^4^ WMH contributes to cognitive decline and have a crucial role in vascular cognitive impairment and dementia and in Alzheimer's disease.^1^, ^4^ However, the underlying neurobiological explanations for the relationship between the spatial distribution of WMH and cognitive decline have not yet been elucidated.

“Functional connectome gradient” refers to a pattern of functional connectivity (FC) that represents the hierarchical architecture of brain networks. This hierarchical architecture, characterized by a modular and parallel structure, is a well‐recognized organizational principle that supports information‐processing across different levels of cognitive function.^5^ As a gradient that reflects the distance between the connectivity patterns of brain regions across the primary sensory network and the transmodal default‐mode network (DMN), the principal connectome gradient has been identified to capture the cognitive spectrum from direct perception and action to increasingly abstract cognition.^6^, ^7^ Recent advancements in noninvasive techniques have allowed researchers to explore the hierarchical architecture of the brain's functional connectome in vivo.^6^, ^8^ In particular, resting‐state functional MRI (rs‐fMRI) has been increasingly used to explore network hierarchy changes and their clinical relevance in various disorders such as Alzheimer's disease,^9^ autism spectrum disorder,^10^ schizophrenia,^11^ and major depressive disorder.^12^

Findings of previous studies have suggested that functional alterations of regional brain activity and connectivity involving the sensory network^13^ and transmodal DMN contribute to WMH‐related cognitive decline.^14^, ^15^, ^16^ However, the main focus of these studies was only on the influence of discrete boundaries of the network and functional abnormalities on WMH‐related cognitive decline; the changes in hierarchical architecture as WMH progresses and their relationship with cognitive decline have not been explored. Exploring the correlation among regional WMH, functional connectome gradient, and cognitive decline could provide new insights into the mechanisms underlying WMH‐related cognitive decline.

In this study, we constructed a network hierarchical architecture in 758 individuals with WMH, based on rs‐fMRI, by using a gradient decomposition framework. We hypothesized that the primary‐to‐transmodal gradient in functional brain networks is altered with WMH progresses and may have a mediation role in WMH‐related cognitive decline. We investigated (1) whether functional connectome gradient would be associated with regional WMH volume and specific cognitive domains, and (2) whether it would mediate the relationship between regional WMH and cognition.

## 2 MATERIALS AND METHODS

### 2.1 Participants

We used data from the Registration of Cerebral Small Vessel Disease, which is an ongoing observational prospective population‐based cohort study conducted at Nanjing Drum Tower Hospital. The aforementioned study focuses on the pathogenesis and key techniques in the clinical evaluation of CSVD (Registration number: ChiCTR‐OOC‐17010562). The study was approved by the Nanjing Drum Tower Hospital Research Ethics Committee. Only sporadic cases of CSVD were included in this study. The definition of WMH of presumed vascular origin was based on previously reported neuroimaging standards.^17^

The inclusion criteria for the WMH participants were as follows: (1) age 50 years or older; (2) presence of mild to severe WMH of presumed vascular origin on MRI (WMH was diagnosed independently and unanimously by two radiologists who visually evaluated the MRI sequences without knowledge of the participants' clinical profiles); and (3) free of dementia.

The exclusion criteria included: (1) a history of ischemic stroke with an infarct size larger than 1.5 cm in diameter or cardiogenic cerebral embolism; (2) cerebral hemorrhage; (3) no recent small subcortical infarctions (infarctions presented high signal in diffusion‐weighted imaging were designated as a recent event); (4) internal carotid artery or vertebral artery stenosis (>50%); (5) WMH due to immune‐mediated demyelinating disease (e.g., multiple sclerosis, neuromyelitis optica, acute disseminated encephalomyelitis); (6) leukodystrophy and genetic leukoencephalopathy (e.g., leuko‐axonopathies, CADASIL, CARASIL); (7) other neurological disease (e.g., Parkinson's disease, epilepsy, or brain tumor); (8) psychiatric disease (e.g., major depressive disorder, schizophrenia, autistic, or bipolar affective disorder); (9) systemic diseases, such as cancer, shock, or anemia; and (10) prominent impairments of audition or vision.

**FIGURE 1:**
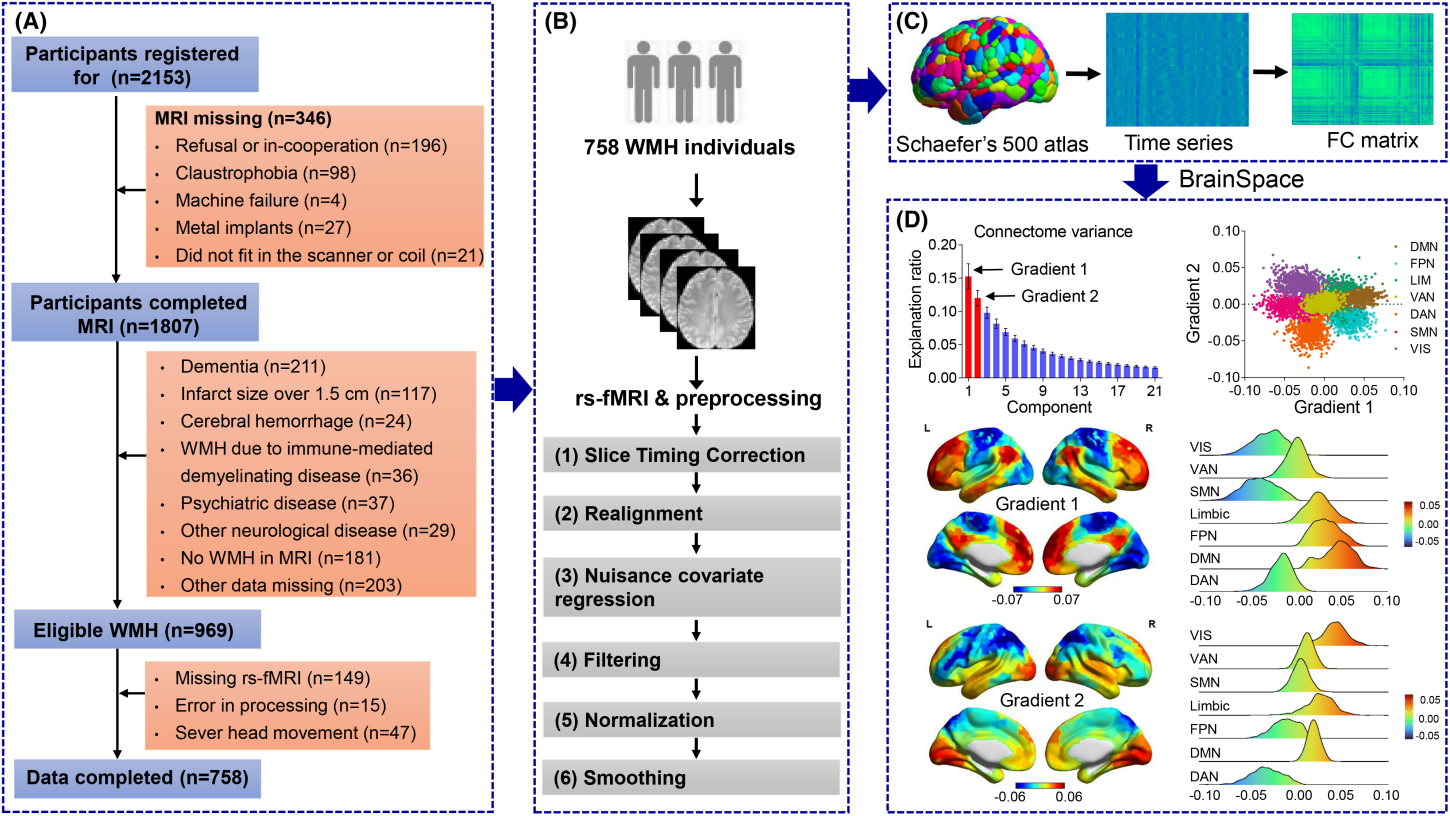
Diagram of the recruitment of WMH participants and the construction of the functional connectome gradient. (A) The flowchart of the study population. (B) Preprocessing of functional images using a standard pipeline. (C) Construction of the FC matrix by calculating Pearson's correlation between time courses of each pair of brain nodes (i.e., regions), defined using Schaefer's 500 Atlas. (D) Connectome gradients in WMH populations. DAN, dorsal attention network; DMN, default‐mode network; FC, functional connectivity; FPN, frontoparietal network; rs‐fMRI, resting‐state functional MRI; SMN, sensorimotor network; VAN, ventral attention network; VIS, visual network; WMH, white matter hyperintensity.

Overall, from January 2017 to December 2022, 2153 participants aged 50 years or older were registered for the CSVD study at the Nanjing Drum Tower Hospital (Figure 1A). Of these, 346 participants had lost MRI data for the following reasons: (1) 196 participants refused to undergo MRI; (2) 98 participants had claustrophobia; (3) four participants had failed MRI scans because of machine failure; (4) 27 participants had metal implants; and (5) 21 participants could not withstand the scanner or coil. Therefore, 1807 individuals successfully finished MRI scanning and 838 of them were further excluded for the following reasons: (1) dementia (*n* = 211); (2) infarcts greater than 1.5 cm in diameter (*n* = 117); (3) cerebral hemorrhage (*n* = 24); (4) WMH due to an immune‐mediated demyelinating disease (*n* = 36); (5) psychiatric disease (*n* = 37); (6) other neurological diseases (*n* = 29); (7) no WMH (*n* = 181); and (8) missed other data (*n* = 203).

Of the 969 eligible WMH participants with MRI data, 149 participants had missing rs‐fMRI data, 15 participants had preprocessing errors, and 47 participants had severe head movements (>3 mm of translation or >3° of rotation). The final subjects of eligible WMH with effective data were 758. Neuropsychological assessment and MRI acquisition were conducted within a time window of 1 month. All participants gave signed informed consent in accordance with the Declaration of Helsinki and underwent detailed clinical evaluations.

### 2.2 Neuropsychological assessment

The Mini‐Mental State Examination (MMSE) was used to identify dementia.^18^ Patients with MMSE scores lower than education‐adjusted norms were defined as having dementia (the cut‐off value was <18 for illiterate participants, <21 for participants with 1–6 years of education, and <25 for participants with >6 years of education). Patients with dementia were excluded from the study. Together with the MMSE, the Montreal Cognitive Assessment (MoCA) was used to evaluate general cognition. The composite score was used to represent performance in specific cognitive domains. The raw test scores were first converted into standardized *Z*‐scores. The composite score of each cognitive domain was then evaluated using the average *Z*‐score of the corresponding subitems. In particular, memory was assessed by using the Wechsler Memory Scale‐Visual Reproduction‐Delayed Recall test and the Auditory Verbal Learning Test‐Long Delayed Recall test. Visuospatial function was assessed by using the clock‐drawing test and visual reproduction copy. Executive function (EF) was derived from the Trail Making Test B (TMT‐B) and Stroop Color and Word Test‐C (SCWT‐C). Information‐processing speed (IPS) was derived from the TMT‐A, SCWT‐A, and SCWT‐B. The EF and IPS deteriorated as the corresponding test scores increased. To facilitate understanding (i.e., a higher score indicating better cognitive performance), we converted the test score to a minus value.

### 2.3 Volume quantification of the brain and WMH

The total intracranial volume (TIV) was automatically obtained using Statistical Parametric Mapping 8 (SPM8; http://www.fil.ion.ucl.ac.uk/spm), based on three‐dimensional (3D)‐T1‐weighted images. Lesion Segmentation Toolbox version 2.0.15 (www.statistical‐modelling.de/lst.html) for SPM 12 (http://www.fil.ion.ucl.ac.uk/spm) was used to semi‐automatically quantify the WMH volume, based on 3D‐T1‐weighted and FLAIR images. The detailed procedures have been described in our previous study.^19^ In brief, the Lesion Growth Algorithm function was applied at a threshold of *κ* = 0.15 and the resulting individual binary lesion map. The threshold *κ* = 0.15 was determined by three experienced neuroradiologists (D.Y., LL.H., and YT.M., with 6, 6, and 5 years of experience in neurovascular imaging, respectively). The anatomical atlases were then inversely normalized to the native space of each binary lesion map to extract the summed WMH volumes in the frontal, temporal, parietal, and occipital lobes (Hammers Atlas 56).^20^ The WMH volume (in milliliters) was defined as the voxel size multiplied by the total number of voxels labeled as lesions.^21^

### 2.4 Preprocessing of rs‐fMRI and construction of the connectome gradient

Functional images were preprocessed using a standard pipeline (Figure 1B) in the toolbox for data processing and analysis of brain imaging (DPARSF; V3.2; www.restfmri.net) and the SPM12 toolkits (www.fil.ion.ucl.ac.uk/spm). Detailed preprocessing procedures are described in our previous study.^16^ Connectome gradient analysis was performed using the MATLAB toolbox for BrainSpace (https://brainspace.readthedocs.io/en/latest/), a compact and flexible toolbox that implements a wide variety of approaches to build macroscale gradients from neuroimaging and connectome data.^8^ For each individual, we first constructed an FC matrix by calculating Pearson's correlation between the time courses of each pair of brain regions and then Z‐transformed all FCs by using Fisher's *r*‐to‐*z* transformation (Figure 1C). Brain regions were defined by using Schaefer's 500 Atlas, which divides brain regions into seven functional systems, based on Yeo's seven‐network parcellation.^22^, ^23^ The top 10% of the connections of each node were then retained, and the cosine similarity between each pair of nodes was computed. Diffusion map embedding,^24^ a nonlinear dimensionality reduction algorithm, was applied to capture the gradient components that explained the variance in the connectivity pattern of the functional connectome. The resulting gradient maps were further aligned across individuals by using iterative Procrustes rotation. This alignment process was repeated 100 times. For each individual, we further evaluated the following global gradient measures: (1) gradient range, defined as the difference between the greatest positive and negative values of the given gradient^10^ (wider range indicates greater differentiation in the encoded connectivity pattern between the regions localized at the gradient ends) and (2) gradient variation, defined as the variance of the given gradient^25^ (greater variation reflects higher heterogeneity in the connectivity structure across regions). In addition, we calculated the relative distance between the subnetworks.^26^ The relative distance between subnetworks was quantified as the difference between the gradient values of the subnetworks. The empirical gradient scores were transformed into ranks of the gradient scores before calculating the gradient difference. The relative distances between pairs of subnetworks captured their interrelationships.

### 2.5 Statistical analysis

WMH volume was log10‐transformed, after adding a constant of 0.01 to all values to avoid log transformation of zero values (some brain lobes may not have WMH). All continuous variables were tested for normality by observing whether the data points on the Probability–Probability Plot basically coincide with the diagonal. According to the results of the test of normality, these continuous variables were presented as the mean ± the standard deviation or as the median (interquartile range [IQR]). All categorical variables were presented as the integer (percentage).

#### 2.5.1 Partial correlation analysis

Partial correlation analysis was used to investigate correlations between regional WMH volume, global gradient measures, and cognition. All statistical procedures were performed using SPSS software (version 22.0; IBM Corporation, Armonk, NY, USA). A value of *p* < 0.05 was statistically significant. Correlations of WMH volume with the relative distance between paired subnetworks and regional gradient measures were assessed using GRETNA v2.0 (https://www.nitrc.org/projects/gretna/). Bonferroni correction was used to control for multiple comparisons of the relative distance analysis (*p* < 0.05/21 was significant). False discovery rate (FDR) correction (*q* = 0.05) was used to control for multiple comparisons of regional gradient analysis. All analyses were adjusted for age, sex, years of education, and TIV.

#### 2.5.2 Mediation analysis

Mediation analysis was conducted to explore whether the primary‐to‐transmodal gradient mediated the association between WMH volume and cognition, after adjusting for age, sex, years of education, and TIV. The bias‐corrected 95% confidence interval (CI) for the mediating effect was calculated by using bootstrapping (*k* = 5000 samples) in PROCESS for SPSS 22.0 (IBM Corporation). The mediating effect was considered statistically significant if the 95% CI did not contain the value 0.

#### 2.5.3 Sensitivity analysis

First, to explore whether the secondary gradient, that is, the DAN‐to‐visual gradient, influenced the results of the main analyses, we reanalyzed all results by additionally regressing out gradient range and variance of the DAN‐to‐visual gradient. Second, the mean framewise displacement was used as another covariate to further control for the motion effect on rs‐fMRI connectivity measures. Furthermore, in order to identify whether the presence of vascular risk factors (i.e., hypertension, diabetes, hyperlipemia, and smoking) affected the results, we also applied them as additional covariates in the sensitivity analysis.

## 3 RESULTS

The demographic and clinical characteristics of the study population are shown in Table 1. The study population consisted of 758 WMH individuals with an average age of 66.69 years and 405 (53.4%) men. The median number of years of education was 12 years. The number of illiterate patients and those with no more than 6 years of education in the final cohort was 20 (2.7%) and 109 (14.4%), respectively. The mean total WMH volume was 8.8 mL. WMH in the frontal, parietal, occipital, and temporal lobes accounted for 36.4%, 16.3%, 7.8%, and 10.1%, respectively, of the total WMH. The median MMSE and MoCA scores were 28 and 24, respectively.

**TABLE 1:** Demographic, neuroimaging, and cognitive data of the WMH population.

Characteristic	Value (*n* = 758)
Demographic characteristics	
Age (years)	66.7 ± 8.2
Sex, male (male %)	405 (53.4)
Education (years)^a^	12 (9,15)
Illiterate patients, *n* (%)	20 (2.7%)
Patients with education years no more than 6, *n* (%)	109 (14.4%)
Vascular risk factors	
Hypertension, *n* (%)	442 (58.3%)
Diabetes, *n* (%)	184 (24.3%)
Hyperlipemia, *n* (%)	188 (24.8%)
Smoking, *n* (%)	166 (21.9%)
Neuroimaging	
TIV (mL)	1364.9 ± 119.9
Total WMH volume (mL)	8.8 ± 11.6
Frontal WMH volume, mL (%)^b^	3.5 ± 4.9 (36.4)
Parietal WMH volume, mL (%)^b^	2.3 ± 4.3 (16.3)
Temporal WMH volume, mL (%)^b^	0.8 ± 1.3 (7.8)
Occipital WMH volume, mL (%)^b^	0.7 ± 0.9 (10.1)
Mean framewise displacement (mm)	0.13 ± 0.09
Cognitive function	
MMSE^a^	28 (27,29)
MoCA^a^	24 (21,26)
IPS^c^	0.01 ± 0.76
Executive function^d^	0.03 ± 0.78
Memory^e^	0.03 ± 0.79
Visuospatial function^f^	0.10 ± 0.75
The first connectome gradient	
Gradient range	0.18 ± 0.03
Gradient variance	0.05 ± 0.01
The second connectome gradient	
Gradient range	0.16 ± 0.02
Gradient variance	0.04 ± 0.01

*Note*: Descriptive statistics were presented as the mean ± standard deviation (SD) or number (percentage) unless otherwise specified.

Abbreviations: IPS, information‐processing speed; MMSE, the mini mental state examination, MoCA, the Montreal cognitive assessment; TIV, Total brain volume; WMH, white matter hyperintensity.

^a^ Data are medians, with interquartile range in parentheses.

^b^ Data are the mean ± standard deviation (SD), with its account for total WMH in parentheses.

^c^ IPS was available in 679 (89.6%) participants.

^d^ Executive function was available in 630 (83.1%) participants.

^e^ Memory was available in 645 (85.1%) WMH participants.

^f^ Visuospatial function was available in 578 (76.3%) participants.

The first two functional connectome gradients are shown in Figure 1D. The first gradient with the sensorimotor network (SMN) and DMN as two anchors of the axes (top right of Figure 1D, *x*‐axis) constituted 15.3% of the total connectivity variance (top left of Figure 1D). It was organized along a gradual axis from the primary SMN to the transmodal DMN (middle of Figure 1D), which is consistent with previous observations of the connectome gradient in healthy adults.^6^ The second gradient constituted an additional 12.0% of the total connectivity variance (top left of Figure 1D), with the dorsal attention network (DAN) at the bottom of the axes and the visual network (VIS) at the top of the axes (top right of Figure 1D, *y*‐axis) distinguishing between DAN and VIS (bottom of Figure 1D). Owing to the association of the primary‐to‐transmodal gradient with the neuronal microstructure and cognitive function,^27^ the present study primarily focused on its correlation with WMH and cognitive function.

### 3.1 Correlation of regional WMH and the primary‐to‐transmodal gradient with cognitive function

**FIGURE 2:**
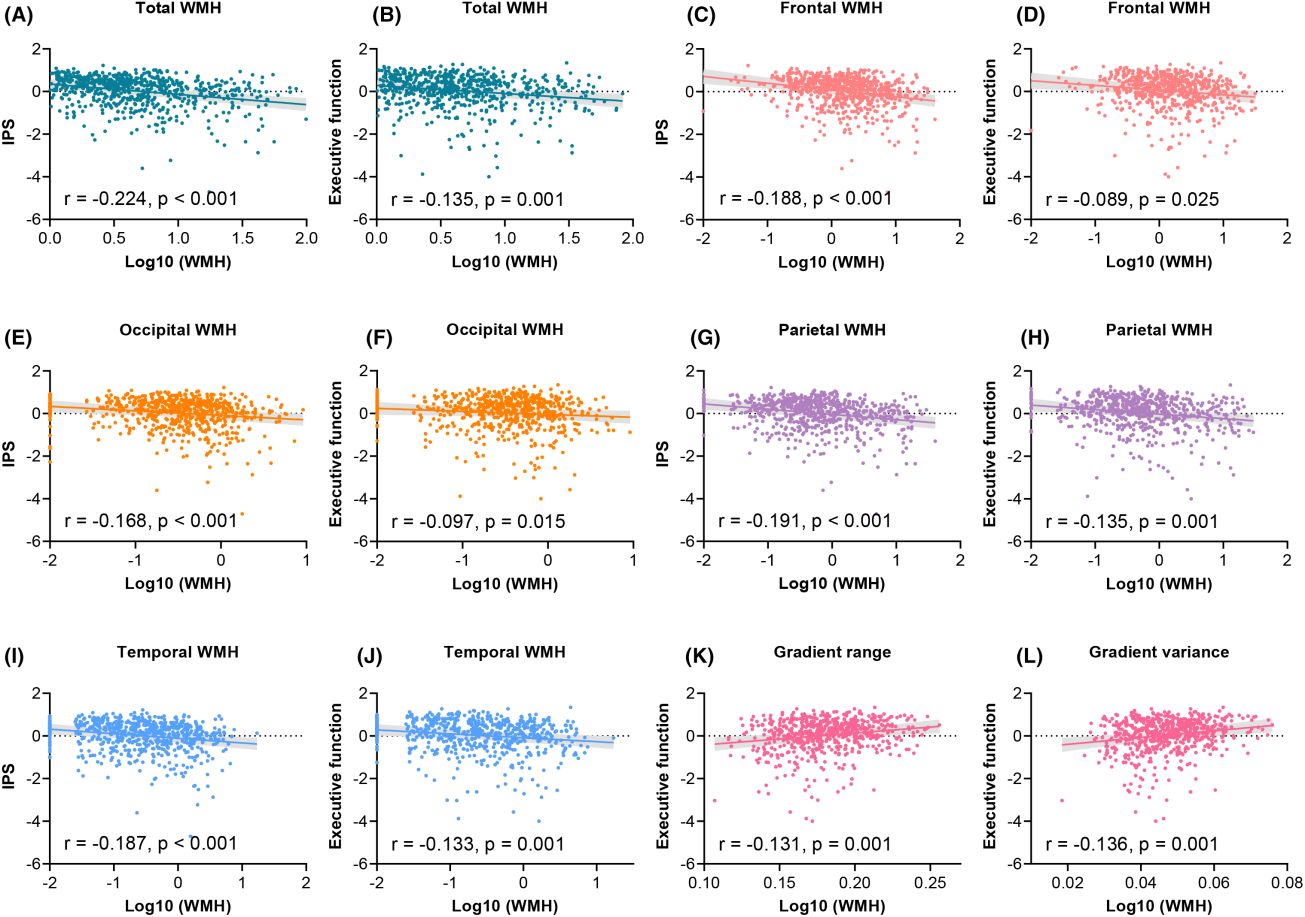
Correlations of regional WMH and global gradient measures of the primary‐to‐transmodal gradient with cognitive function. (A, B) The total WMH was negatively associated with IPS (A) and executive function (B). (C, D) The frontal WMH was negatively associated with IPS (C) and executive function (D). (E, F) The occipital WMH was negatively associated with IPS (E) and executive function (F). (G, H) The parietal WMH was negatively associated with IPS (G) and executive function (H). (I, J) The temporal WMH was negatively associated with IPS (I) and executive function (J). (K, L) The gradient range (K) and variance (L) were positively associated with executive function. IPS, information‐processing speed; WMH, white matter hyperintensity.

As shown in Figure 2, the total WMH volume was negatively associated with IPS (*r* = −0.224, *p* < 0.001, Figure 2A) and EF (*r* = −0.135, *p* = 0.001, Figure 2B), after adjusting for age, sex, years of education, and TIV. Similar correlations were found for the frontal, parietal, temporal, and occipital WMH (for all, *p* < 0.05, Figure 2C–J). For the primary‐to‐transmodal gradient, we observed that the global gradient range (*r* = 0.131, *p* = 0.001, Figure 2K) and variance (*r* = 0.136, *p* = 0.001, Figure 2L) were only positively associated with EF.

### 3.2 Correlation of the regional WMH with the primary‐to‐transmodal gradient

In this section, we calculated the correlations between regional WMH and the primary‐to‐transmodal gradient at three different levels (i.e., whole‐brain, subnetwork, and regional levels), after adjusting for age, sex, years of education, and TIV.

**FIGURE 3:**
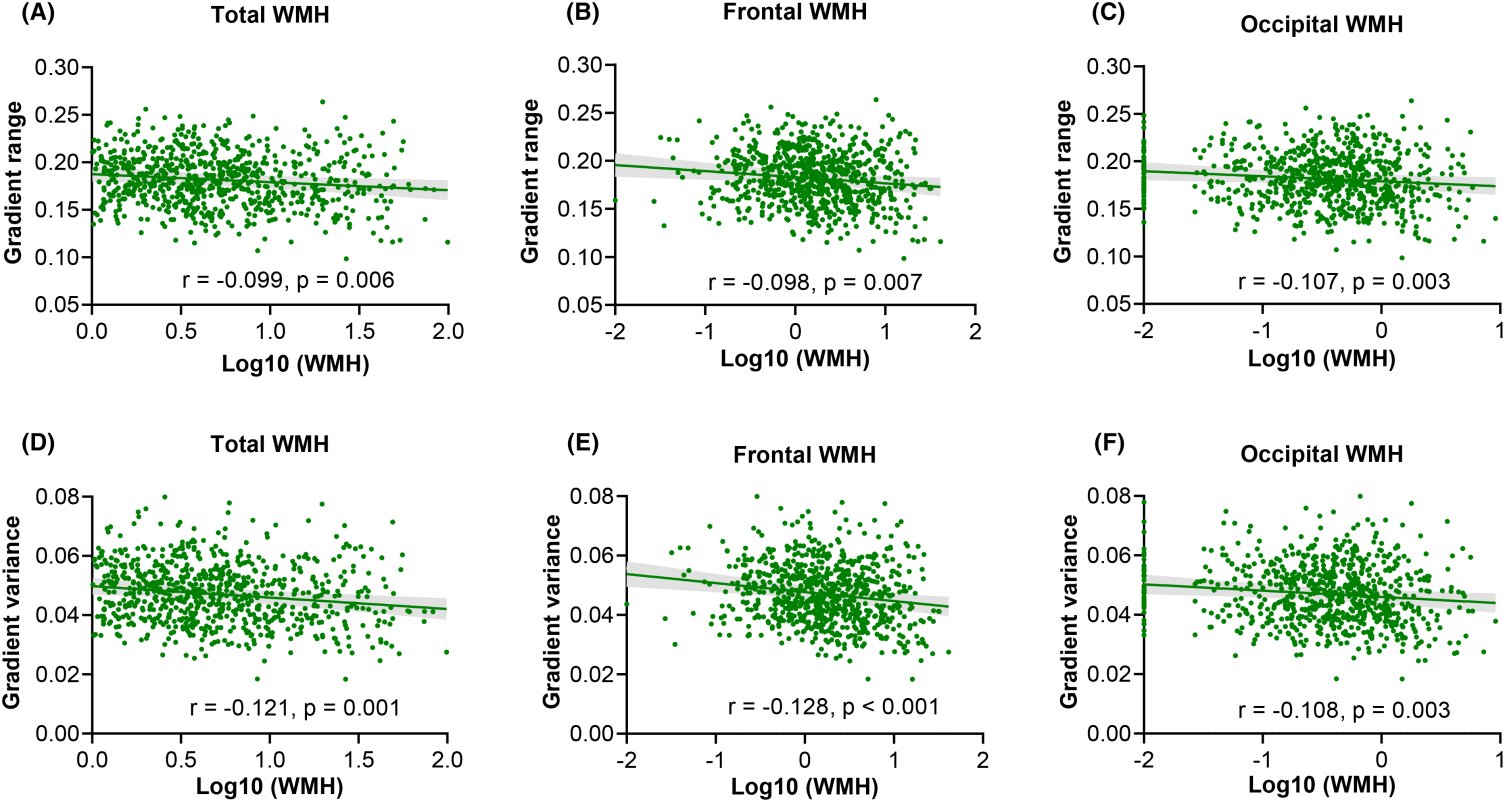
Correlations between regional WMH and global gradient measures of the primary‐to‐transmodal gradient. (A–C) The total (A), frontal (B), and occipital (C) WMH were negatively associated with gradient range, respectively. (D–F) The total (D), frontal (E), and occipital (F) WMH were negatively associated with gradient variance, respectively. WMH, white matter hyperintensity.

First, the correlations between WMH volume and global gradient measures were explored. As shown in Figure 3, WMH was negatively associated with the gradient range and variance. In particular, the higher the total WMH volume, the narrower the gradient range (*r* = −0.099, *p* = 0.006, Figure 3A) and the smaller the gradient variance (*r* = −0.121, *p* = 0.001, Figure 3D). Similarly, higher frontal and occipital WMH volumes corresponded to a narrower gradient range (frontal WMH: *r* = −0.098 and *p* = 0.007, Figure 3B; occipital WMH: *r* = −0.107 and *p* = 0.003, Figure 3C) and smaller gradient variance (frontal WMH: *r* = −0.128 and *p* < 0.001, Figure 3E; occipital WMH: *r* = −0.108 and *p* = 0.003, Figure 3F).

**FIGURE 4:**
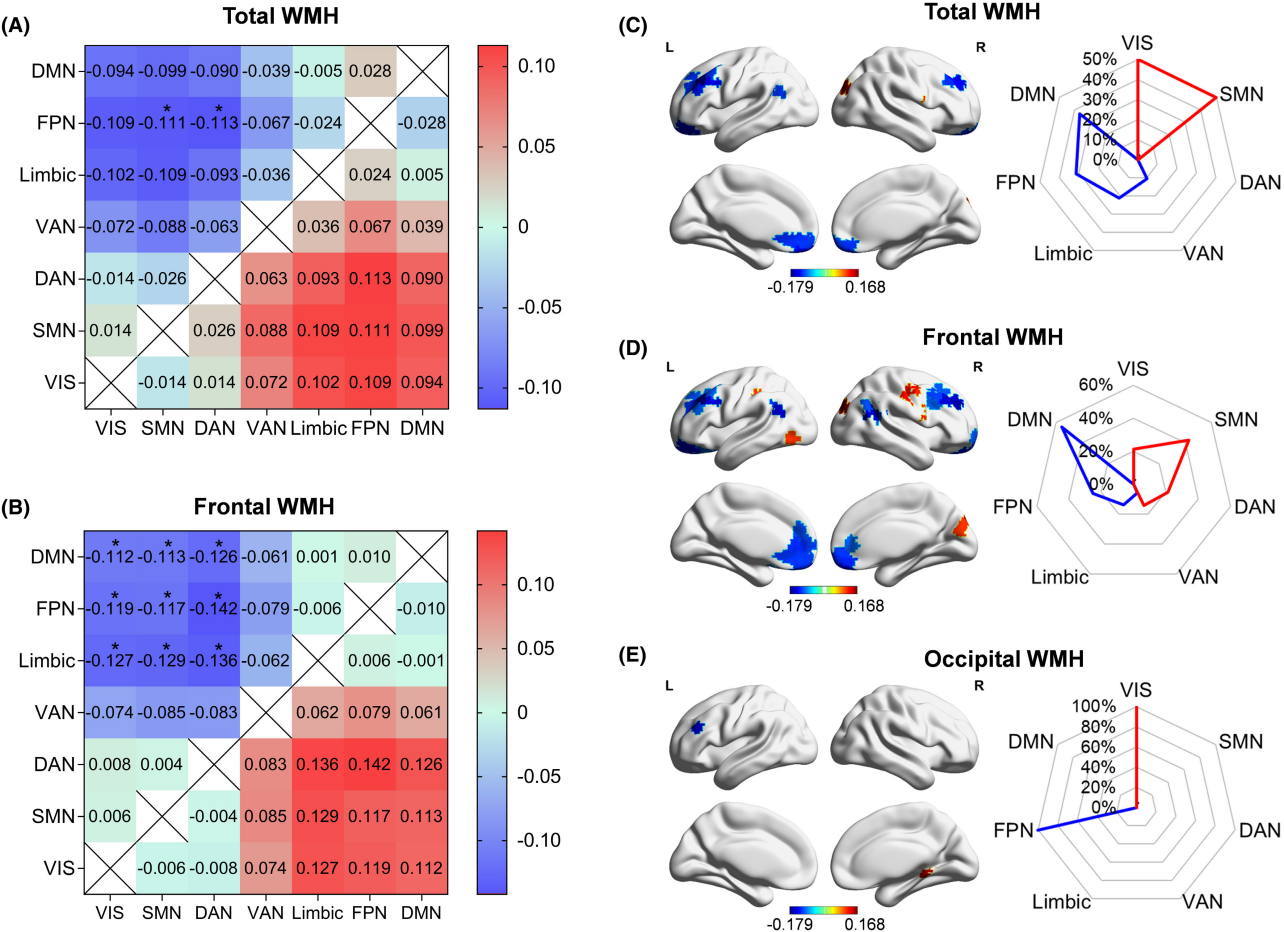
Correlations between regional WMH and the primary‐to‐transmodal gradient at subnetwork and regional levels. (A, B) Correlations of the total WMH (A) and frontal WMH (B) with the relative distance between pair subnetworks [Bonferroni correction (*indicated *p* < 0.05/21)]. (C–E) Correlations of the total (C), frontal (D), and occipital (E) WMH with the regional gradient score and its distribution in different subnetworks (FDR correction, *q* = 0.05, the positive and negative correlations were presented in red and blue colors, respectively). DAN, dorsal attention network; DMN, default‐mode network; FDR, false discovery rate; FPN, frontoparietal network; SMN, sensorimotor network; VAN, ventral attention network; VIS, visual network; WMH, white matter hyperintensity.

Second, we explored the correlations between WMH volume and the relative distance between subnetworks in the primary‐to‐transmodal gradient. The results showed that the total WMH was negatively associated with the relative distance from the SMN and ventral attention network (VAN) to the frontoparietal network (FPN) (FPN‐SMN: *r* = −0.111; FPN‐VAN: *r* = −0.113; all *p* < 0.05/21; Figure 4A). Further correlation analyses of the WMH in each lobe with the relative distance showed that only the frontal WMH was negatively associated with the relative distance from the VIS/SMN/DAN to DMN/FPN/limbic (for all, *p* < 0.05/21; Figure 4B). These findings suggested that the separation of the primary‐to‐transmodal gradient decreased with the progression of WMH.

Finally, we investigated the correlations between WMH volume and regional gradient score of the primary‐to‐transmodal gradient. As shown in Figure 4C, we observed that the brain regions in which the gradient score was positively correlated with the total WMH were in the SMN (50%) and VIS (50%), whereas the brain regions in which the gradient score was negatively correlated with the total WMH were mostly distributed in high‐order subnetworks (i.e., DMN [36.8%], FPN [31.6%], and limbic regions [21.1%]). For the frontal WMH (Figure 4D), the brain regions in which the gradient score was positively correlated with the frontal WMH were primarily in the low‐order subnetworks (i.e., SMN [42.9%], VIS [21.4%], and DAN [21.4%]), whereas the brain regions in which the gradient score was negatively correlated with the frontal WMH were mostly distributed in the high‐order subnetworks (i.e., DMN [55.6%], FPN [25.0%], and limbic regions [13.9%]). Figure 4E displays that only two brain regions existed in which the gradient score was correlated with the occipital WMH: one region was in the VIS (positive correlation) and the other region was in the FPN (negative correlation). Detailed information on the pertinent brain regions is provided in Table S1.

Of note, we did not find any prominent correlations between the parietal/temporal WMH and the primary‐to‐transmodal gradient, indicating that the connectome gradient alterations were primarily specific to the frontal and occipital WMH.

### 3.3 Mediation effect of the primary‐to‐transmodal gradient on the relationship between WMH and cognitive decline

**FIGURE 5:**
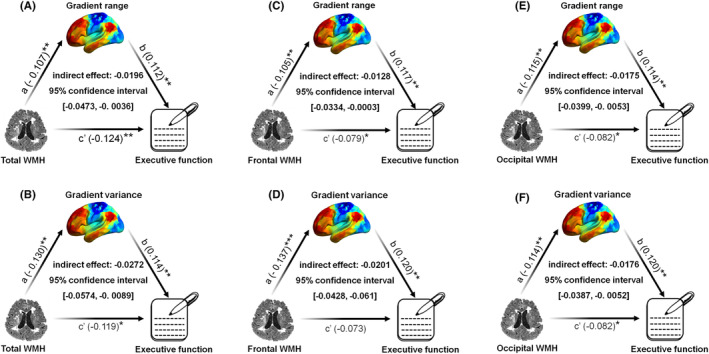
Mediation model of WMH on executive function through the primary‐to‐transmodal gradient. (A, B) The gradient range (A) and variance (B) mediated the relationship between the total WMH and executive function. (C, D) The gradient range (C) and variance (D) mediated the relationship between the frontal WMH and executive function. (E, F) The gradient range (E) and variance (F) mediated the relationship between the occipital WMH and executive function (*p < 0.05). WMH, white matter hyperintensity.

To further explore whether the primary‐to‐transmodal gradient was in the potential causal pathway of the association between WMH and specific cognitive domains, mediation models were constructed for WMH volume, global gradient range/variance, and cognitive performance. We found that the global gradient range (indirect effect = −0.0196; 95% CI [−0.0473, −0.0036]; Figure 5A) and variance (indirect effect = −0.0272; 95% CI [−0.0574, −0.0089]; Figure 5B) significantly mediated the relationship between total WMH and EF. A comparable mediating effect on the relationship between frontal/occipital WMH and EF was also observed (i.e., 95% CI did not contain the value 0), as shown in Figure 5C–F.

### 3.4 Sensitivity analyses

Regardless of whether range/variance of the DAN‐to‐visual gradient, the mean framewise displacement, and vascular risk factors were additionally regressed, the main results of this study did not change. First, correlations of range/variance of the primary‐to‐transmodal gradient with regional WMH and cognition were similar to the results of this study (Table S2). Second, correlations between regional WMH and the primary‐to‐transmodal gradient at subnetwork and regional levels were consistent with this study (Figures S1 and S2). Third, the mediating effects of gradient range/variance on the relationship between frontal/occipital WMH and EF remain unaltered (Table S3).

## 4 DISCUSSION

The aim of this study was to determine the interrelations among the spatial distribution of WMH, functional connectome gradient, and domain‐specific cognition in a large population with mild to severe WMH. The results showed that the frontal and occipital WMH volumes were negatively associated with EF, IPS, as well as the range and variance of the primary‐to‐transmodal gradient. The global gradient range and variance of the primary‐to‐transmodal gradient partly bridged the connection between the frontal/occipital WMH and EF. These findings provide valuable insights into the role of the primary‐to‐transmodal gradient in WMH‐related cognitive decline.

Researchers have widely found that network hierarchy guides the flow of information across the brain cortex, where signals are promoted to become increasingly bound to other information and then transformed into more abstract cognition.^28^ This fundamental principle has been observed across multiple subsystems, including sensory, motor, and higher‐order transmodal networks.^29^ In the present study, a larger WMH volume corresponded to a narrower gradient range and a smaller gradient variance, indicating less differentiated connectivity patterns between the primary and transmodal areas with the progression of WMH. With regard to the primary‐to‐transmodal gradient, the brain regions of the DMN were geometrically located in the cortex with maximal spatial distances from the primary sensorimotor system.^6^ This factor ensured a complete processing route from the mapping of concrete stimuli to the integration of abstract conceptions.^30^ In the information‐processing hierarchy, the primary cortex is at the bottom end to receive immediate environmental inputs, process raw sensory signals, and then transfer specialized representations to transmodal areas.^6^ Further analysis revealed that the gradient range and variance were positively associated with EF in WMH individuals. This finding was consistent with the notion that a long distance between the primary and transmodal areas may protect the transmodal regions from interference from immediate external inputs and facilitate the formation of abstract cognitive functions. In addition, mediation analysis revealed that a narrower gradient range and smaller variance, to a certain extent, mediated the decline in EF related to WMH. From a pathological mechanistic viewpoint, we speculated that WMH may shorten the distance between the primary and transmodal areas, disrupt information transmission and integration, and lead to cognitive decline.

Our regional gradient analysis revealed that when the WMH burden (especially frontal WMH) was more severe, the gradient score of the brain regions in low‐order subnetworks (i.e., SMN and VIS) and DAN was higher and the gradient score of the brain regions distributed in high‐order subnetworks (DMN, FPN, limbic regions) was lower. In addition, the analysis of the relative distance between subnetworks revealed that the more severe the frontal WMH burden, the shorter the relative distance from the low‐ to high‐order subnetworks in the primary‐to‐transmodal gradient. These results together indicated that the frontal WMH may disrupt the separation pattern from low‐ to high‐order networks. This finding again confirmed the hypothesis that a shorter distance from the primary to transmodal areas may contribute to WMH‐related cognitive decline.

Importantly, with regard to spatial distribution of WMH, the present study originally revealed that only the frontal and occipital WMH were associated with the primary‐to‐transmodal gradient. The frontal and occipital lobes play distinct and critical roles in brain function including high‐level cognitive functions (e.g., decision‐making, planning, and executive function) and visual information processing.^31^, ^32^ The realization of these functions depends on the integrality of an intricate set of short‐ and long‐range connections that guarantee direct access to sensory information and control over regions dedicated to planning and motor execution.^33^ The pathology of WMH generally reflects loss of axons and myelin, myelin pallor, and gliosis.^34^ Our previous works have revealed that WMH could disrupted microstructure of nerve fibers and impaired structural network properties.^35^, ^36^ It has been reported that the functional connectome can be anatomically shaped by the structural connectome.^37^ Given the extensive connectivity of the frontal and occipital lobes with other brain regions. We speculated that WMH in these two lobes may lead to weakened or disrupted structural connections, thereby affecting the primary‐to‐transmodal gradient. However, it remains to be further elaborated through diffusion tensor imaging or relevant animal models for CSVD and cognitive decline.^38^

Several issues should be considered. First, the primary‐to‐transmodal gradient was obtained at the level of 500 brain regions rather than at the voxel level. A high consistency was observed between our gradient patterns (i.e., 500 brain regions) and those used in previous studies (i.e., voxel level); however, further studies should be conducted to validate the main findings in this work at a higher resolution. Second, although the high level of significance in partial correlation analysis, coefficients were small. These small coefficients indicated that the linear correlations were weak in this study, and there were other nonlinear relationships. Future studies should further identify these nonlinear relationships and the mechanisms underlying them. Third, due to the current study concentrated primarily on interrelations among the spatial distribution of WMH, functional gradient measures, and specific cognitive domains, we refrained from stratifying WMH patients with mild, moderate, and severe WMH. Nevertheless, the severity of WMH in the population varies widely, future research that further classifies WMH severity and delves into its differential impact on functional gradient and cognitive decline in WMH subgroups will be crucial for understanding its full clinical and pathological spectrum. Lastly, this study was cross‐sectional. No causal inferences could therefore be determined. We will continue to recruit new participants and conduct a follow‐up with them to validate our findings.

## 5 CONCLUSIONS

In conclusion, we mapped the network hierarchy patterns of individuals with WMH and found that the WMH volume, primary‐to‐transmodal gradient, and cognitive decline were interrelated. The decreased differentiation of connectivity patterns between the primary and transmodal areas explains the decline in EF caused by the frontal and occipital WMH. These findings provide new insights into the neuropathological mechanisms underlying WMH‐related cognitive decline.

## AUTHOR CONTRIBUTIONS

YX designed this study. DY wrote the manuscript and statistically analyzed the MRI and cognitive data. YT and ZHK helped analyze MRI data. ZXZ, LLH, YTM, and LMRT helped analyze cognitive data. CLM, ZQH, YC, PFS, and BZ helped collected clinical data. XLZ and YX reviewed and revised the manuscript.

## FUNDING INFORMATION

This research was supported by the National Natural Science Foundation of China (82130036, 81920108017), the STI2030‐Major Projects (2022ZD0211800), the Jiangsu Province Key Medical Discipline (ZDXK202216), the Key Research and Development Program of Jiangsu Province of China (BE2020620), and the Postgraduate Research & Practice Innovation Program of Jiangsu Province (SJCX23_0670).

## CONFLICT OF INTEREST STATEMENT

Xu, Yun is an Editorial Board member of CNS Neuroscience and Therapeutics and a co‐author of this article. To minimize bias, she was excluded from all editorial decision‐making related to the acceptance of this article for publication.

## Supporting information


Tables S1–S3.

Figures S1–S2.

## Data Availability

Data described in the article will be made available upon request by bona fide researchers for specified scientific purposes via contacting the corresponding authors.
