# Supplementary material for: Connectome gradient dysfunction contributes to white matter hyperintensity‐related cognitive decline
Source: CNS Neurosci Ther. 2024 Jul 12;30(7):e14843. doi: 10.1111/cns.14843 (PMC11245402; doi:10.1111/cns.14843)
Supplement: Supplementary file 1 — Tables S1–S3. [file CNS-30-e14843-s002.docx]

**Supplementary Tables**

**Supplementary Table S1 Brain regions showing significant association between regional gradient score of the primary-to-transmodal gradient and WMH volume**

| **ROI Label** | **ROI Name** | **Coordinates** | | | **P value** | **r** |
| --- | --- | --- | --- | --- | --- | --- |
|  |  | **R** | **A** | **S** |  |  |
| **Total WMH** | | | | | | |
| 128 | 7Networks_LH_SalVentAttn_PFCl_1 | -28 | 44 | 30 | 0.001 | -0.118 |
| 141 | 7Networks_LH_Limbic_OFC_3 | -6 | 46 | -22 | <0.001 | -0.134 |
| 142 | 7Networks_LH_Limbic_OFC_4 | -16 | 48 | -20 | <0.001 | -0.147 |
| 166 | 7Networks_LH_Cont_OFC_1 | -30 | 52 | -12 | <0.001 | -0.132 |
| 172 | 7Networks_LH_Cont_PFCl_6 | -42 | 36 | 28 | <0.001 | -0.127 |
| 174 | 7Networks_LH_Cont_PFCl_8 | -46 | 18 | 36 | <0.001 | -0.139 |
| 175 | 7Networks_LH_Cont_PFCl_9 | -36 | 32 | 38 | <0.001 | -0.182 |
| 201 | 7Networks_LH_Default_Par_4 | -48 | -56 | 24 | 0.002 | -0.115 |
| 211 | 7Networks_LH_Default_PFC_5 | -4 | 56 | -10 | 0.002 | -0.115 |
| 212 | 7Networks_LH_Default_PFC_6 | -6 | 36 | -8 | 0.001 | -0.119 |
| 228 | 7Networks_LH_Default_PFC_22 | -20 | 40 | 42 | 0.001 | -0.117 |
| 231 | 7Networks_LH_Default_PFC_25 | -40 | 18 | 50 | 0.002 | -0.115 |
| 283 | 7Networks_RH_Vis_33 | 22 | -86 | 30 | <0.001 | 0.163 |
| 300 | 7Networks_RH_SomMot_15 | 62 | -2 | 12 | <0.001 | 0.130 |
| 384 | 7Networks_RH_SalVentAttn_PFCl_1 | 28 | 44 | 34 | <0.001 | -0.133 |
| 401 | 7Networks_RH_Limbic_OFC_4 | 10 | 48 | -22 | 0.001 | -0.122 |
| 404 | 7Networks_RH_Limbic_OFC_7 | 8 | 62 | -14 | 0.002 | -0.112 |
| 438 | 7Networks_RH_Cont_PFCl_15 | 40 | 34 | 38 | 0.001 | -0.119 |
| 440 | 7Networks_RH_Cont_PFCl_17 | 32 | 32 | 40 | <0.001 | -0.133 |
| 475 | 7Networks_RH_Default_PFCdPFCm_1 | 6 | 46 | -12 | 0.001 | -0.117 |
| 485 | 7Networks_RH_Default_PFCdPFCm_11 | 22 | 40 | 38 | <0.001 | -0.143 |
| **Frontal WMH** | | | | | | |
| 8 | 7Networks_LH_Vis_8 | -44 | -70 | -8 | 0.002 | 0.112 |
| 63 | 7Networks_LH_SomMot_24 | -38 | -18 | 42 | 0.003 | 0.109 |
| 94 | 7Networks_LH_DorsAttn_Post_8 | -44 | -32 | 44 | 0.002 | 0.110 |
| 128 | 7Networks_LH_SalVentAttn_PFCl_1 | -28 | 44 | 30 | 0.001 | -0.121 |
| 140 | 7Networks_LH_Limbic_OFC_2 | -24 | 22 | -20 | 0.005 | -0.102 |
| 141 | 7Networks_LH_Limbic_OFC_3 | -6 | 46 | -22 | 0.001 | -0.122 |
| 142 | 7Networks_LH_Limbic_OFC_4 | -16 | 48 | -20 | <0.001 | -0.144 |
| 166 | 7Networks_LH_Cont_OFC_1 | -30 | 52 | -12 | <0.001 | -0.135 |
| 172 | 7Networks_LH_Cont_PFCl_6 | -42 | 36 | 28 | 0.004 | -0.105 |
| 174 | 7Networks_LH_Cont_PFCl_8 | -46 | 18 | 36 | <0.001 | -0.144 |
| 175 | 7Networks_LH_Cont_PFCl_9 | -36 | 32 | 38 | <0.001 | -0.179 |
| 201 | 7Networks_LH_Default_Par_4 | -48 | -56 | 24 | <0.001 | -0.145 |
| 204 | 7Networks_LH_Default_Par_7 | -58 | -54 | 30 | 0.001 | -0.120 |
| 211 | 7Networks_LH_Default_PFC_5 | -4 | 56 | -10 | 0.001 | -0.117 |
| 212 | 7Networks_LH_Default_PFC_6 | -6 | 36 | -8 | 0.001 | -0.119 |
| 217 | 7Networks_LH_Default_PFC_11 | -6 | 46 | 4 | 0.001 | -0.123 |
| 218 | 7Networks_LH_Default_PFC_12 | -6 | 60 | 6 | 0.001 | -0.116 |
| 222 | 7Networks_LH_Default_PFC_16 | -6 | 46 | 18 | 0.002 | -0.111 |
| 228 | 7Networks_LH_Default_PFC_22 | -20 | 40 | 42 | 0.003 | -0.108 |
| 229 | 7Networks_LH_Default_PFC_23 | -26 | 28 | 44 | 0.004 | -0.105 |
| 231 | 7Networks_LH_Default_PFC_25 | -40 | 18 | 50 | 0.004 | -0.105 |
| 281 | 7Networks_RH_Vis_31 | 6 | -82 | 26 | 0.002 | 0.113 |
| 283 | 7Networks_RH_Vis_33 | 22 | -86 | 30 | <0.001 | 0.168 |
| 300 | 7Networks_RH_SomMot_15 | 62 | -2 | 12 | 0.001 | 0.126 |
| 303 | 7Networks_RH_SomMot_18 | 54 | -10 | 36 | 0.002 | 0.115 |
| 305 | 7Networks_RH_SomMot_20 | 54 | -16 | 40 | 0.001 | 0.124 |
| 307 | 7Networks_RH_SomMot_22 | 52 | -12 | 50 | 0.001 | 0.122 |
| 309 | 7Networks_RH_SomMot_24 | 46 | -10 | 50 | <0.001 | 0.128 |
| 359 | 7Networks_RH_DorsAttn_FEF_1 | 40 | -4 | 52 | <0.001 | 0.132 |
| 364 | 7Networks_RH_DorsAttn_PrCv_2 | 58 | 8 | 32 | 0.002 | 0.112 |
| 374 | 7Networks_RH_SalVentAttn_PrC_1 | 54 | 0 | 46 | 0.001 | 0.117 |
| 383 | 7Networks_RH_SalVentAttn_FrOperIns_9 | 60 | 8 | 18 | 0.002 | 0.113 |
| 384 | 7Networks_RH_SalVentAttn_PFCl_1 | 28 | 44 | 34 | <0.001 | -0.165 |
| 401 | 7Networks_RH_Limbic_OFC_4 | 10 | 48 | -22 | <0.001 | -0.140 |
| 404 | 7Networks_RH_Limbic_OFC_7 | 8 | 62 | -14 | 0.001 | -0.124 |
| 425 | 7Networks_RH_Cont_PFCl_2 | 22 | 64 | -6 | 0.004 | -0.105 |
| 437 | 7Networks_RH_Cont_PFCl_14 | 40 | 14 | 32 | 0.005 | -0.103 |
| 438 | 7Networks_RH_Cont_PFCl_15 | 40 | 34 | 38 | <0.001 | -0.150 |
| 439 | 7Networks_RH_Cont_PFCl_16 | 44 | 18 | 44 | 0.002 | -0.115 |
| 440 | 7Networks_RH_Cont_PFCl_17 | 32 | 32 | 40 | <0.001 | -0.162 |
| 453 | 7Networks_RH_Default_Par_1 | 56 | -46 | 14 | 0.003 | -0.108 |
| 454 | 7Networks_RH_Default_Par_2 | 48 | -60 | 20 | <0.001 | -0.143 |
| 455 | 7Networks_RH_Default_Par_3 | 52 | -50 | 20 | <0.001 | -0.161 |
| 456 | 7Networks_RH_Default_Par_4 | 54 | -44 | 26 | <0.001 | -0.128 |
| 457 | 7Networks_RH_Default_Par_5 | 48 | -60 | 32 | 0.003 | -0.109 |
| 475 | 7Networks_RH_Default_PFCdPFCm_1 | 6 | 46 | -12 | 0.001 | -0.125 |
| 477 | 7Networks_RH_Default_PFCdPFCm_3 | 8 | 54 | -4 | 0.001 | -0.119 |
| 479 | 7Networks_RH_Default_PFCdPFCm_5 | 8 | 42 | 6 | 0.005 | -0.103 |
| 485 | 7Networks_RH_Default_PFCdPFCm_11 | 22 | 40 | 38 | <0.001 | -0.152 |
| 487 | 7Networks_RH_Default_PFCdPFCm_13 | 24 | 26 | 46 | 0.003 | -0.108 |
| **Occipital WMH** | | | | | | |
| 172 | 7Networks_LH_Cont_PFCl_6 | -42 | 36 | 28 | <0.001 | -0.152 |
| 266 | 7Networks_RH_Vis_16 | 18 | -44 | -2 | <0.001 | 0.158 |

Partial correlation analysis was applied after adjusting for age, sex, years of education, and TIV. FDR correction (*q* = 0.05) was used to control multiple comparisons. Cont = control; DorsAttn = dorsal attention network; FEF = frontal eye fields; FrOperIns = frontal operculum insula; LH = left hemisphere; OFC = orbital frontal cortex; PrC = precentral; PrCv = precentral ventral; SomMot = somatomotor; post = posterior; Par = parietal; PFCd = dorsal prefrontal cortex; PFC = prefrontal cortex; PFCl = lateral prefrontal cortex; PFCdPFCm = dorsal prefrontal cortex medial prefrontal cortex; RH = right hemisphere; ROI = region of interest; Temp = temporal; Vis = visual cortex

**Supplementary Table S2 Correlation of range/variance of the primary-to-transmodal gradient with regional WMH and cognition in sensitivity analysis**

| **Items** | **Gradient range** | | **Gradient variance** | |
| --- | --- | --- | --- | --- |
|  | **r** | **P value** | **r** | **P value** |
| **WMH volume** |  |  |  |  |
| Total WMH | **-0.099** | **0.007** | **-0.116** | **0.001** |
| Frontal WMH | **-0.100** | **0.006** | **-0.132** | **<0.001** |
| Occipital WMH | **-0.115** | **0.002** | **-0.112** | **0.002** |
| Parietal WMH | -0.047 | 0.195 | -0.061 | 0.094 |
| Temporal WMH | -0.050 | 0.171 | -0.046 | 0.210 |
| **Cognitive function** |  |  |  |  |
| MMSE | -0.018 | 0.633 | -0.019 | 0.602 |
| MoCA | -0.017 | 0.646 | -0.029 | 0.428 |
| IPS | -0.011 | 0.772 | -0.007 | 0.852 |
| Executive function | **0.118** | **0.003** | **0.124** | **0.002** |
| Memory | -0.025 | 0.554 | -0.024 | 0.559 |
| Visuospatial function | -0.021 | 0.616 | -0.066 | 0.115 |

Adjusted for age, sex, years of education, TIV, range/variance of the DAN-to-visual gradient, mean framewise displacement, and vascular risk factors. IPS = information processing speed; MMSE = the mini mental state examination; MoCA = the Montreal cognitive assessment; WMH = white matter hyperintensity. Bold indicated *P* < 0.05.

**Supplementary Table S3 Mediation model of WMH on executive function through the primary-to-transmodal gradient in sensitivity analysis**

| **Items** | | **Path a** | | **Path b** | | **Path c’** | | **Indirect effect (path a·b)** | |
| --- | --- | --- | --- | --- | --- | --- | --- | --- | --- |
|  |  | **Standardized Coefficients** | **P value** | **Standardized Coefficients** | **P value** | **Standardized Coefficients** | **P value** | **β** | **95%CI** |
| **Total WMH** | **Gradient range** | **-0.099** | **0.007** | **0.106** | **0.009** | **-0.129** | **0.001** | **-0.019** | **-0.048, -0.005** |
|  | **Gradient variance** | **-0.125** | **0.002** | **0.108** | **0.007** | **-0.131** | **0.001** | **-0.026** | **-0.056, -0.008** |
| **Frontal WMH** | **Gradient range** | **-0.100** | **0.006** | **0.111** | **0.006** | **-0.082** | **0.041** | **-0.013** | **-0.034, -0.002** |
|  | **Gradient variance** | **-0.126** | **0.001** | **0.114** | **0.005** | -0.078 | 0.054 | **-0.020** | **-0.043, -0.006** |
| **Occipital WMH** | **Gradient range** | **-0.115** | **0.002** | **0.106** | **0.008** | **-0.090** | **0.026** | **0.018** | **-0.040, -0.005** |
|  | **Gradient variance** | **-0.112** | **0.002** | **0.113** | **0.005** | **-0.090** | **0.022** | **-0.018** | **-0.038, -0.005** |

Adjusted for age, sex, years of education, TIV, range/variance of the DAN-to-visual gradient, mean framewise displacement, and vascular risk factors. CI = confidence interval. WMH = white matter hyperintensity. Bold indicated *P* < 0.05 or 95% confidence interval did not contain the value 0.
