# Supplementary material for: Connectome gradient dysfunction contributes to white matter hyperintensity‐related cognitive decline
Source: CNS Neurosci Ther. 2024 Jul 12;30(7):e14843. doi: 10.1111/cns.14843 (PMC11245402; doi:10.1111/cns.14843)
Supplement: Supplementary file 2 — Figures S1–S2. [file CNS-30-e14843-s001.docx]

**Supplementary Figures**

**Supplementary Figure S1**

**Fig. S1** Correlation between WMH volume and the relative distance between pair subnetworks of the primary-to-transmodal gradient in sensitivity analysis (Bonferroni correction, * indicated *P* < 0.05/21). (A) Correlation between the total WMH volume and the relative distance between pair subnetwork. (B) Correlation between the frontal WMH volume and the relative distance between pair subnetwork. DAN = dorsal attention network; DMN = default-mode network; FC = functional connectivity; FPN = frontoparietal network; rs-fMRI = resting-state functional MRI; SMN = sensorimotor network; VAN = ventral attention network; VIS = visual network; WMH = white matter hyperintensity.

**Supplementary Figure S2**

**Fig. S2** Correlation between WMH volume and regional gradient score of the primary-to-transmodal gradient in sensitivity analysis (FDR-corrected, *q* = 0.05). Positive and negative correlations were presented as red and blue colors, respectively. (A) Correlation of the total WMH with regional gradient score and its distribution in different subnetworks. (B) Correlation of the frontal WMH with regional gradient score and its distribution in different subnetworks. (C) Correlation of the occipital WMH with regional gradient score and its distribution in different subnetworks. DAN = dorsal attention network; DMN = default-mode network; FC = functional connectivity; FPN = frontoparietal network; rs-fMRI = resting-state functional MRI; SMN = sensorimotor network; VAN = ventral attention network; VIS = visual network; WMH = white matter hyperintensity.
